# Growth and hormone profiling in children with congenital melanocytic naevi

**DOI:** 10.1111/bjd.14091

**Published:** 2015-11-17

**Authors:** R. Waelchli, J. Williams, T. Cole, M. Dattani, P. Hindmarsh, H. Kennedy, A. Martinez, S. Khan, R.K. Semple, A. White, N. Sebire, E. Healy, G. Moore, V.A. Kinsler

**Affiliations:** ^1^ Department of Paediatric DermatologyGreat Ormond Street Hospital for ChildrenLondonWC1N 3JHU.K.; ^2^ Childhood Nutrition Research CentreUCL Institute of Child HealthLondonU.K.; ^3^ MRC Centre of Epidemiology for Child HealthUCL Institute of Child HealthLondonU.K.; ^4^ Department of Genetics and Genomic MedicineUCL Institute of Child HealthLondonU.K.; ^5^ Department of Paediatric EndocrinologyGreat Ormond Street Hospital for ChildrenLondonWC1N 3JHU.K.; ^6^ Faculty of Medical and Human SciencesUniversity of ManchesterManchesterU.K.; ^7^ Wellcome Trust‐MRC Institute of Metabolic ScienceUniversity of CambridgeCambridgeU.K.; ^8^ Department of Paediatric HistopathologyGreat Ormond Street Hospital for ChildrenLondonWC1N 3JHU.K.; ^9^ Department of DermatopharmacologySir Henry Wellcome LaboratoriesUniversity of SouthamptonSouthamptonU.K.

## Abstract

**Background:**

Multiple congenital melanocytic naevi (CMN) is a rare mosaic RASopathy, caused by postzygotic activating mutations in *NRAS*. Growth and hormonal disturbances are described in germline RASopathies, but growth and hormone status have not previously been investigated in individuals with CMN.

**Objectives:**

To explore premature thelarche, undescended testes, and a clinically abnormal fat distribution with CMN through prospective endocrinological assessment of a cohort of subjects with CMN, and a retrospective review of longitudinal growth of a larger group of patients with CMN from outpatient clinics (which included all subjects in the endocrinological assessment group).

**Patients and methods:**

Longitudinal growth in a cohort of 202 patients with single or multiple CMN was compared with the U.K. National Child Measurement Programme 2010. Forty‐seven children had hormonal profiling including measurement of circulating luteinizing hormone, follicle‐stimulating hormone, thyroid stimulating hormone, adrenocorticotrophic hormone, growth hormone, prolactin, pro‐opiomelanocortin, estradiol, testosterone, cortisol, thyroxine, insulin‐like growth factor‐1 and leptin; 10 had oral glucose tolerance testing 25 had dual‐energy X‐ray absorptiometry scans for body composition.

**Results:**

Body mass index increased markedly with age (coefficient 0·119, SE 0·016 standard deviation scores per year), at twice the rate of the U.K. population, due to increased adiposity. Three per cent of girls had premature thelarche variant and 6% of boys had persistent undescended testes. Both fat and muscle mass were reduced in areas underlying large naevi, resulting in limb asymmetry and abnormal truncal fat distribution. Anterior pituitary hormone profiling revealed subtle and variable abnormalities. Oral glucose tolerance tests revealed moderate–severe insulin insensitivity in five of 10, and impaired glucose tolerance in one.

**Conclusions:**

Interpersonal variation may reflect the mosaic nature of this disease and patients should be considered individually. Postnatal weight gain is potentially related to the underlying genetic defect; however, environmental reasons cannot be excluded. Naevus‐related reduction of fat and muscle mass suggests local hormonal or metabolic effects on development or growth of adjacent tissues, or mosaic involvement of these tissues at the genetic level. Premature thelarche and undescended testes should be looked for, and investigated, as for any child.

## Abstract 2

**What's already known about this topic?**

- CMN are caused by postzygotic mutations in the gene *NRAS* in the majority of cases, classifying it within the group of mosaic RASopathies.
- Other germline and mosaic RASopathies are known to have growth and hormonal abnormalities.
- No studies have been done on growth or endocrinology in children with CMN.

**What does this study add?**

- Average body mass index increases markedly with age compared with the normal population; this is due to increased adiposity, and can be associated with insulin insensitivity.
- Premature thelarche variant and persistent undescended testes are not infrequent findings, but puberty appears to develop normally.
- Both fat and muscle mass can be reduced in areas underlying large naevi, resulting in asymmetry.

**Linked Comment:** Millington, Br J Dermatol 2015; 173: 1366–67.

Congenital melanocytic naevi (CMN) are moles that are present from birth. These can be isolated cutaneous lesions, single or multiple (defined as two or more CMN at birth), or when multiple can be associated with extracutaneous abnormalities, then termed CMN syndrome. Extracutaneous associations described thus far are characteristic facial features,^1^ and a wide range of neurological abnormalities detectable on magnetic resonance imaging (MRI),^2^, ^3^ seen in approximately 20% of children with more than one CMN at birth.^4^ Molecular studies have established that approximately 80% of cases of multiple CMN or CMN syndrome are caused by mosaicism for oncogenic mutations in codon 61 of *NRAS*,^5^ classifying the majority as a mosaic RASopathy. Primary melanoma can arise within the skin or central nervous system (CNS), more commonly in those with a severe cutaneous phenotype,^6^ and this malignant complication is associated with further genetic events.^5^

Many RASopathies manifest abnormalities of pre‐ or postnatal growth, or of endocrine dysfunction. For example, neurofibromatosis type 1 is characterized by macrocephaly,^7^ early puberty and growth failure,^8^ and Costello syndrome babies have a higher than average birthweight^9^ often with hypoglycaemia.^10^ Moreover, cardio–facio–cutaneous syndrome, Noonan syndrome and Noonan syndrome with multiple lentigines are associated with postnatal short stature, and complex subtle endocrine abnormalities are seen frequently in Noonan syndrome.^11^, ^12^ The molecular mechanisms behind such variation in growth and endocrine abnormalities in the RASopathies is not understood; however, the MAPK pathway is known to be involved in metabolic signalling.^13^

Several clinical observations directed our attention to metabolic and endocrine function in children with CMN (Fig. 1): firstly, the clinically well‐recognized decrease in bulk of subcutaneous tissues under large CMN, hitherto thought to be fat atrophy but never formally studied; secondly, the previously undescribed presentation of premature thelarche variant in a series of young girls with CMN; thirdly, the occurrence of undescended testes in some babies with bathing trunk naevi (i.e. affecting the genital area).

**Figure 1:**
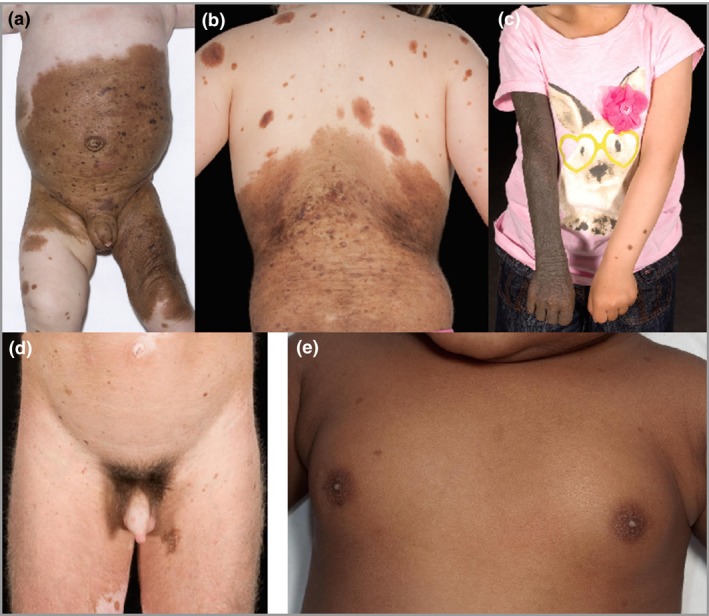
Clinical images of children with multiple congenital melanocytic naevi showing (a–c) lack of bulk of underlying tissues associated with overlying naevus: (a) decreased girth but not length of left leg; (b) lower abdomen; (c) decreased girth but not length of right arm; (d) characteristic pubic hair development in a child of 7 years, without other secondary sexual development; (e) premature thelarche in a child of 1 year, not geographically associated with naevi.

Premature thelarche variant (sometimes termed exaggerated thelarche) is a relatively rare subset of premature secondary sexual development that shares features with both isolated premature thelarche and precocious puberty.^14^ It is characterized biochemically by the dominance of serum concentrations of follicle‐stimulating hormone (FSH) over luteinizing hormone (LH), and clinically by a slight acceleration in growth, the appearance of sparse pubic hair together with breast development, and/or minor increases in uterine measurements,^15^ in the presence of a normal MRI of the CNS. It does not affect final height, unlike true precocious puberty.

Growth and hormone status have not previously been investigated in individuals with CMN. We recruited a cohort of subjects with CMN for prospective endocrinological assessment, and used a larger group of patients with CMN from outpatient clinics (which included all subjects in the endocrinological assessment group) for a retrospective review of longitudinal growth.

## Patients and methods

This study was approved by the Great Ormond Street Hospital for Children (GOSH)/Institute of Child Health Research Ethics Committee and R&D office, and complies with the Declaration of Helsinki protocols.

### Growth study

Patient records were obtained from 202 individuals with CMN seen sequentially in the outpatient department of GOSH between December 2009 and February 2011, and all recorded growth measurements were analysed retrospectively. Data sets collected comprised weight, height and occipito–frontal circumference (OFC) measurements, CMN phenotype, neurological status (clinical and radiological) and family history. CMN phenotype was classified as in Table S1 (see Supporting Information) as previously published.^16^, ^17^ Weight and height were measured by a paediatric nurse, and OFC was measured by a single observer (V.K.) in more than 95% of cases. OFC at the oldest age measured was used in all analyses to allow for any settling of head circumference after birth. One patient was removed from OFC analysis as there was an unrelated reason for microcephaly (postnatal hypoglycaemic injury).

The median number of measurement sets from each patient was 3·0 (range 1–14). The mean age of the children at the time of analysis was 5·2 years (SEM 0·4). The time covered by the study was 468 years, with 765 sets of postnatal growth measurements obtained for 184 individuals (88 males). Birthweight and delivery details were available for 157 children.

### Endocrine study

#### Patient group

All families in the growth study with children aged 1 year or more were invited to take part in the endocrine study and 47 gave written consent to participate. The lower age limit of 1 year was to avoid any influence of maternal hormones from pregnancy, and to allow the use of topical anaesthetic cream for venepuncture.

Venous blood samples were obtained from 47 children (29 females, 18 males) of mean age 6·3 years (SEM 0·6) between 09·30 and 11·30 h, and analysed for the following concentrations: pro‐opiomelanocortin (POMC), adrenocorticotrophic hormone (ACTH), thyroid stimulating hormone (TSH), LH, FSH, growth hormone (GH), prolactin, insulin‐like growth factor 1 (IGF‐1), cortisol, estradiol, testosterone and thyroxine. Blood for the measurement of POMC was collected into ethylenediamine tetraacetic acid tubes containing aprotinin onto ice. Serum and plasma were collected by standard methods within 2 h of venepuncture, and frozen until required. All hormones were measured in the GOSH diagnostic biochemistry laboratory other than POMC, which was measured using a validated immunoassay.^18^

#### Control group

In the absence of published data on systemic plasma POMC concentrations in children, and poorly defined reference ranges for leptin concentrations, we recruited a control group specifically for these measurements. We obtained written consent for 19 children (10 females) having laser surgery for single port‐wine stains with no other abnormalities, and blood samples were taken between the hours of 09·00 and 11·00 h, comparable with the CMN group. The mean age of this group was 5·2 years, comparable with the CMN group. Blood collection and handling was the same as for the patient group. For all other hormone concentrations the hospital reference ranges were used.

### Oral glucose tolerance tests

Ten children with multiple CMN underwent an oral glucose tolerance test (OGTT) following the standard hospital protocol. Patient ages, BMI, and detailed results are shown in Table S2 (see Supporting Information); all were postpubertal. Briefly, the patients fasted from midnight the night before, an intravenous cannula was inserted the following morning, and then baseline insulin and glucose (as well as IGF‐1, IGF‐BP3, inhibin, LH, FSH and vitamin D) were measured half an hour after cannula insertion. A standard dose of glucose was then ingested orally, and insulin and glucose measured at 30, 60 and 90 min.

### Dual‐energy X‐ray absorptiometry scanning

A whole‐body dual‐energy X‐ray absorptiometry (DXA) scan to examine body composition was performed on 25 children with CMN using Lunar Prodigy instrumentation (GE Medical Systems, Slough, U.K.) in conjunction with software v.12·1. All scans were performed by one operator (J.W.) with the subject wearing light indoor clothing. The scan provides values for whole‐body and regional (trunk and limbs) fat mass, bone mineral content and nonosseous lean. Values were converted to standard deviation score (SDS) using the LMS method^19^ from reference data collected using the same instrument.^20^ The precision of soft tissue analysis for a Lunar DPX‐L instrument (regarded by the manufacturer, GE Medical Systems, as similar to our own model) established by repeated measurements of humans on 4 successive days was reported as 1% for fat‐free mass and 2% for fat mass.^21^

### Statistical analysis

Growth measurements including birthweight were expressed as SDS relative to the British 1990 growth reference, using the LMSgrowth add‐in for Excel.^19^, ^22^, ^23^ The age trend in body mass index (BMI) SDS within individuals was modelled with random‐effects regression. For comparison the prevalence of being overweight for years 1 and 6 in the National Child Measurement Programme 2010 was obtained^24^ and expressed as an annual trend in SDS. The methods of initiation of labour and of delivery were compared with the most recently available U.K. national statistics,^25^ using the chi‐square test with Yates's correction. Mean POMC and leptin levels between patient and control groups were compared by independent *t*‐test.

## Results

### Birthweight, occipito–frontal circumference, delivery and gestation results

Information on type of birth, gestation and birthweight was available for 157 children, 149 born at full term (37–42 weeks; 61 males) and eight born prematurely. Birthweight distribution for full‐term infants was normal, mean and median 3·31 kg (SD 0·47) for females and 3·55 kg (SD 0·44) for males (Fig. S1; see Supporting Information). The mean birthweight SDS for the whole group was −0·02. Mean OFC SDS was 0·17 (Fig. S1; see Supporting Information), and OFC was not associated with either clinical or radiological neurological phenotype.

Data on delivery were compared with the most recent Hospital Episode Statistics data for the U.K. population.^23^, ^25^ Labour was induced in 26% of mothers in the CMN cohort, compared with 8% in the normal U.K. population (*P* < 0·001) (Fig. S1; see Supporting Information). Reasons for induction were failure of contractions to begin, either over 41 weeks' gestation or after spontaneous rupture of membranes (55%), with maternal hypertension the next most common reason (16%).

### Postnatal growth results

**Figure 2:**
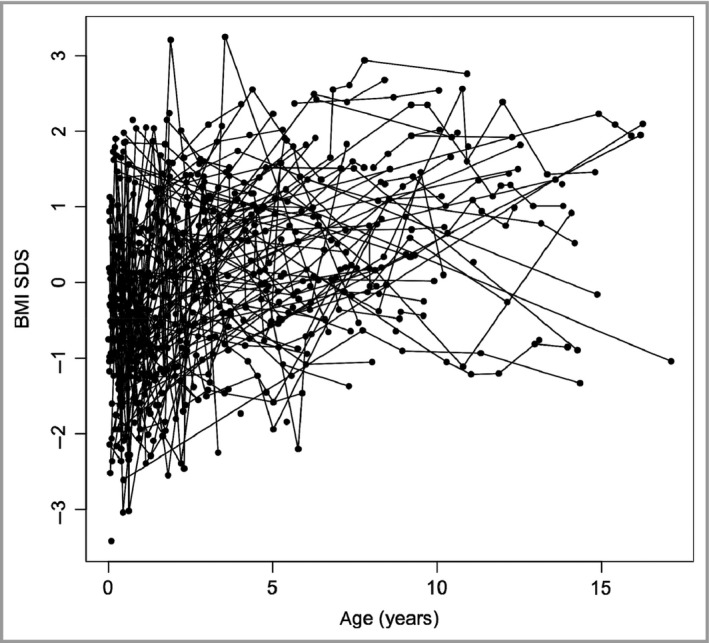
Markedly increasing body mass index (BMI) standard deviation score (SDS) with age in longitudinal growth measurements in the congenital melanocytic naevi patient cohort, analysed by random‐effects regression.

BMI SDS increased significantly with age (Fig. 2), at a rate of 0·119 (SE 0·016 SDS per year; *P* < 0·001). The corresponding National Child Measurement Programme rates for overweight were 23·1% in year 1 (age 5–6 years) and 33·4% in year 6 (age 10–11 years), corresponding to an underlying BMI SDS change from −0·74 to −0·43 over the 5 years, or 0·061 SDS per year for U.K. children as a whole,^24^ close to half the rate of the CMN cohort. The BMI SDS was not associated with the severity of cutaneous phenotype on regression analysis using Projected Adult Size of the largest CMN, or single vs. multiple (more than one at birth) CMN, or total number of CMN as variables.

### Comparison of endocrine study and growth cohorts

The characteristics of the growth cohort and the endocrine study cohort subset were compared with look for bias due to self‐selection based on endocrine abnormalities or severity of CMN phenotype. Cutaneous phenotype profiles were very similar in both these groups (Table S1; see Supporting Information). In addition, there were no significant differences in first‐degree family history rates of all endocrine or thyroid disease between the two groups.

### Circulating hormone levels

Several anterior pituitary hormone concentration abnormalities were noted in the children with CMN (Table 1); most notably LH was undetectable (< 0·2 U L^−1^) in 71% of those tested. The hospital reference ranges for LH were based on Immulite company values, and independent establishment of the reference range in normal children describes a normal distribution within the following ranges: male 0–13 years: < 0·1–4·0, 13–19 years: < 0·1–3·7 L^−1^; female 0–6 years: < 0·1–3·3, 6–11 years: < 0·1–5·0, 11–15 years: < 0·1–13·4, 15–19 years: < 0·1–16·4 L^−1^.^26^ On this basis, one would expect far fewer than 71% to be undetectable. However, it is considered more difficult to interpret suppressed values than raised values, and this observation requires confirmation in future studies. In three patients with premature thelarche variant, the same pattern of undetectable LH was noted, and gonadotrophin stimulation testing revealed FSH dominance over LH, supporting the observation that LH is usually low in these patients.

**Table 1:** Abnormal circulating hormone concentrations for 47 patients in the CMN cohort, with respect to standard Children's Hospital normal ranges. Single ACTH and GH concentrations should be interpreted with caution due to circadian changes

	TSH	ACTH	GH	IGF‐1	PL	LH	FSH	POMC
Concentration low, *n* (%)	0 (0)	10 (22)	5 (11)	5 (24)	6 (14)	32 (71)	5 (11)	0 (0)
Concentration high, *n* (%)	2 (4)	1 (2)	15 (33)	0 (0)	2 (5)	2 (4)	3 (6)	4 (9)
Missing	2	1	1	26	3	2	2	0

CMN, congenital melanocytic naevi; TSH, thyroid stimulating hormone; ACTH, adrenocorticotrophic hormone; GH, growth hormone; IGF‐1, insulin‐like growth factor 1; PL, prolactin; LH, luteinizing hormone; FSH, follicle‐stimulating hormone; POMC, pro‐opiomelanocortin.

Cortisol, estradiol and thyroxine concentrations were normal for age in the CMN group. Isolated GH and ACTH measurements cannot be interpreted because of the cyclical nature of secretion, but these results led us to check IGF‐1 concentrations in those for whom sufficient sample remained. Twenty‐one children had IGF‐1 concentrations measured and six were low, although this did not correlate with low GH measurements; their ages were not significantly different from the median of the group, excluding young age as a reason for low IGF‐1. Mean leptin concentrations were not significantly different from controls: CMN group mean 3·99 ng mL^−1^ SEM 0·84; control group mean 3·02 ng mL^−1^ SEM 0·84. Four patients had POMC concentrations greater than 100 pmol L^−1^, considered to be the cut‐off for diagnosing POMC‐secreting tumours,^27^, ^28^ with none in the control group. The unusually high concentrations did not correlate in any obvious way with the severity of cutaneous phenotype in the patients, and the mean concentration was not significantly different from that of the control group (CMN group mean 29·6 pmol L^−1^ SEM 4·4; control group mean 33·2 pmol L^−1^ SEM 3·7). We assume that these values may be at the extreme of the normal range, or there is the possibility that some factor in the patients' plasma is interfering with the assay. Secretion of POMC by the large number of naevus cells could also explain the high POMC values in the four patients. In the other patients rapid metabolism of POMC to its cleavage product α‐melanocyte‐stimulating hormone (MSH) cannot be excluded. However, despite considerable evaluation of α‐MSH kits, it was not possible to measure it reliably.

### Oral glucose tolerance tests

**Figure 3:**
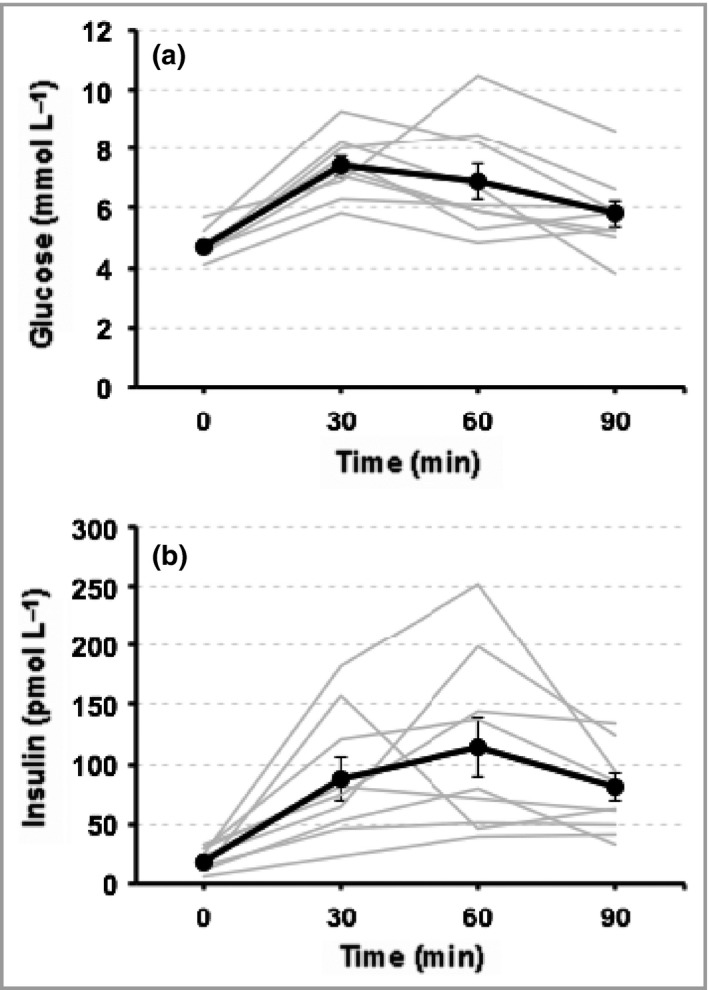
Graphic illustration of change in concentrations of circulating blood (a) glucose and (b) insulin in 10 children with multiple congenital melanocytic naevi, after ingestion of a standardized oral glucose dose. Each coloured line represents a different patient. Raw data is shown in Table S2 (see Supporting Information).

Results for the 10 children with multiple CMN who underwent an OGTT are shown in Fig. 3 and Table S2 (see Supporting Information). Fasting glucose and insulin were within normal limits; however, five of the 10 children demonstrated moderate–severe insulin insensitivity, and one was suggestive of impaired glucose tolerance.

### Dual‐energy X‐ray absorptiometry

**Figure 4:**
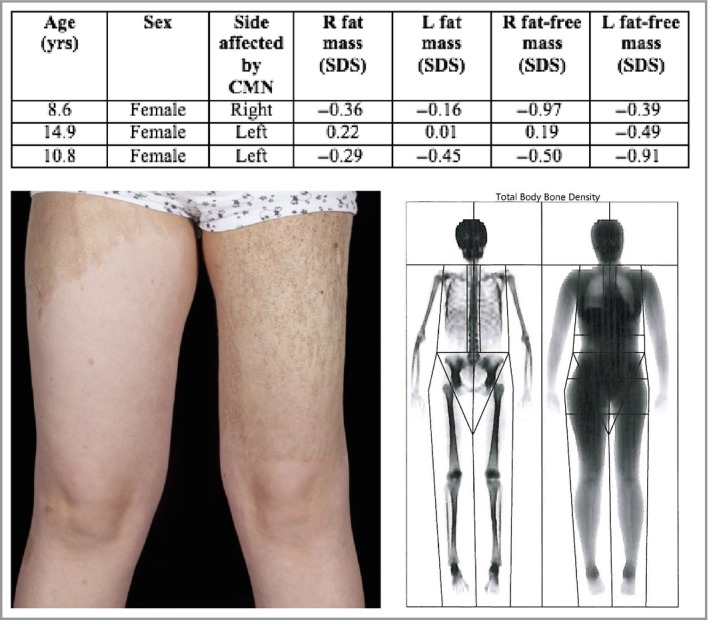
DXA scanning of asymmetric limbs in three patients to quantify asymmetry using SDS derived from a large in‐house control population of normal children,^20^ showing reduction in both fat mass and fat‐free mass (muscle) on the side affected by the naevi (see details in the table). Osseous mass was not reduced. Clinical and DXA images of the one of the patients are shown. CMN, congenital melanocytic naevi; DXA, dual‐energy X‐ray absorptiometry; SDS, standard deviation scores.

Results for the 25 children with multiple CMN who underwent DXA scanning are shown in Fig. 4 and Table S3 (see Supporting Information). Two important conclusions could be drawn: firstly, that in children with increased BMI this increase was attributable to increased adiposity rather than overgrowth of other tissues such as muscle or bone; secondly, not only fat but muscle was reduced underlying large CMN, but no evidence for bony underdevelopment was seen.

### Premature thelarche, undescended testes and early pubic hair growth

On review of our GOSH CMN registry at the time of the collection of hormone levels, at a point when detailed face‐to‐face phenotyping data were available for 224 (101 male) children, 3% of girls with any size of CMN had premature thelarche variant and 6% of boys had at least one undescended testis persistent at the age of 1 year (four unilateral, one bilateral), all of which required surgical correction. Among the girls, none of the largest CMN overlay the breast tissue, and of the boys four had their largest CMN in a bathing trunk distribution but not necessarily including the genital area, and the fifth had the largest CMN covering almost the whole of one leg. Neither premature thelarche variant nor undescended testes was significantly associated with the Projected Adult Size of the largest CMN or with single vs. multiple naevi, or the total number of CMN on logistic regression analysis; however, the numbers involved here are small.

The incidence of unilateral undescended testis persisting to the age of 1 year in the normal population is 1%, but is far commoner in some RASopathies such as Noonan syndrome.^29^, ^30^ Interestingly, the incidence of isolated premature thelarche is associated with increasing BMI, being less than 3% at 8 years of age in those with a normal BMI but significantly higher in those with a BMI greater than the 85th centile,^31^ which may be relevant to our population. However, premature thelarche variant is much rarer, suggesting that the subtle circulating hormonal abnormalities may also have a role to play in individuals with CMN.

Another clinical feature is the very common development of pubic hair at an early age in children where the CMN involves the genital area (Fig. 1d). CMN tissue frequently demonstrates increased hair growth, and therefore this may simply be an exacerbation of the normal hair growth pattern of this lesion, but in an area that is locally primed to be androgen‐responsive. This hypothesis would be supported by the absence of any other secondary sexual characteristics in these children at that point.

## Discussion

This study describes metabolic and hormonal disturbances in children with CMN, namely a marked increase in average postnatal growth rate, variable subtle hormonal changes occasionally accompanied by premature thelarche variant with slow progression to puberty or persistent undescended testes, underdevelopment of fat and muscle underlying large CMN, and a tendency to insulin insensitivity.

Prenatal growth of children with CMN does not appear to be affected by *NRAS* mosaicism, at least as measured by the indices of birthweight and head circumference, echoing the findings in one previous small study.^32^ Interestingly, in 1981 Castilla *et al*. reported an increase in birthweight in postmature infants in a large series of children with CMN.^33^ However, this study was conducted in the cultural context of gestation extending naturally up to 47 weeks, and the key finding was in fact continuing fetal weight gain after 40 weeks' gestation, which is against the normal pattern of weight loss after 40 weeks with a gradually failing placenta. This may be the correlate of our unexpected finding of significantly increased rate of induction of labour, as both factors suggest that the complex endocrine triggers for the end of pregnancy are not occurring. This mirrors the unexplained but consistent pattern of delayed labour in mothers carrying babies with certain other congenital conditions such as anencephaly.

However, looking in more detail at prenatal growth, there is marked underdevelopment of both adipose tissue and skeletal muscle underlying large CMN. This does not measurably affect limb length, while girth is clearly altered. Patients do not usually report any symptoms relating to this feature; however, one patient consistently reports that her thinner leg is weaker when swimming. The underdevelopment of subcutaneous fat and muscle is noticeable from birth, and is therefore primarily a developmental phenomenon; however, the effect is maintained throughout life. Surgical transplant of subcutaneous fat into the thinner buttock and thigh has been used to good cosmetic effect in some of our patients. Interestingly, and in support of this primarily being a prenatal developmental abnormality, the transplanted fat does not appear to be affected by the naevus.

Postnatally the marked increase in BMI, which according to DXA is due to an increase in adiposity rather than overgrowth of muscle or bone, may be related to the psycho‐social effects of significant disfigurement leading to overfeeding or overeating, or decreased levels of physical activity. However, this is not routinely seen with other disfiguring skin conditions in our practice. An alternative is that it is related to the underlying genetic defect, given the known metabolic effects of the MAPK pathway,^13^ and known NRAS expression in adipocytes,^34^ although there is also evidence that MAPK activation inhibits adipogenesis.^35^ However, the interconnected multiple effector pathways downstream of *NRAS* frequently do not behave in predictable ways, and in a mosaic disorder with two populations of cells with different MAPK activations this may be even more complex. Whether *NRAS* is mutated in adipocytes of patients with multiple CMN has not yet been ascertained.

Circulating hormonal abnormalities are subtle and variable, perhaps reflecting the mosaic nature of this condition. Patients should therefore be considered on an individual basis, and if presenting with premature thelarche should have a full workup including brain imaging to exclude non‐CMN‐related causes if LH/FSH are raised. Undescended testes should be managed in the standard way. A possible mechanism underlying these abnormalities could be related to overactivity of the G‐protein function of *NRAS* in many pathways, supported by data from germline RASopathies,^11^ but further molecular work will be required to elucidate the molecular basis.

## Supporting information


**Table S1.** Cutaneous phenotype of the congenital melanocytic naevi (CMN) growth cohort (bold) and the endocrine study cohort (not bold), showing that the two cohorts are phenotypically similar.
**Table S2.** Phenotype and absolute values for 10 patients with congenital melanocytic naevi who underwent oral glucose tolerance test. All concentrations other than glucose and insulin were measured only at baseline. ×0, ×30, ×60 and ×90 represent values at time zero, 30, 60 and 90 min, respectively, from ingestion of standard glucose dose.
**Table S3.** Raw data from whole‐body composition analysis using DXA scanning of 25 patients with congenital melanocytic naevi. One patient was too young for DXA SDS reference cohort data. SDS, standard deviation score; Wt, weight; Ht, height; DXA, dual‐energy X‐ray absorptiometry; BMC, bone mineral content; FM, fat mass; FFM, fat‐free mass.
**Fig S1.** Upper panel: histograms showing absolute birthweight (kg) in females and males and occipito–frontal head circumference standard deviation score (SDS) for full‐term infants in the cohort with congenital melanocytic naevi (CMN). Lower panel: comparison of method of onset of labour and delivery between the CMN cohort and national statistics 2009–10. Numbers for induction of labour do not include elective caesarean section. *P*‐values are for Yate's chi‐square test.
