# Supplementary material for: Growth and hormone profiling in children with congenital melanocytic naevi
Source: Br J Dermatol. 2015 Nov 17;173(6):1471–8. doi: 10.1111/bjd.14091 (PMC4737097; doi:10.1111/bjd.14091)
Supplement: Supplementary file 1 — Table S1. Cutaneous phenotype of the congenital melanocytic naevi (CMN) growth cohort (bold) and the endocrine study cohort (not bold), showing that the two cohorts are phenotypically similar. Table S2. Phenotype and absolute values for 10 patients with congenital melanocytic naevi who underwent oral glucose tolerance test. All concentrations other than glucose and insulin were measured only at baseline. ×0, ×30, ×60 and ×90 represent values at time zero, 30, 60 and 90 min, respectively, from ingestion of standard glucose dose. Table S3. Raw data from whole‐body composition analysis using DXA scanning of 25 patients with congenital melanocytic naevi. One patient was too young for DXA SDS reference cohort data. SDS, standard deviation score; Wt, weight; Ht, height; DXA, dual‐energy X‐ray absorptiometry; BMC, bone mineral content; FM, fat mass; FFM, fat‐free mass. Fig S1. Upper panel: histograms showing absolute birthweight (kg) in females and males and occipito–frontal head circumference standard deviation score (SDS) for full‐term infants in the cohort with congenital melanocytic naevi (CMN). Lower panel: comparison of method of onset of labour and delivery between the CMN cohort and national statistics 2009–10. Numbers for induction of labour do not include elective caesarean section. P‐values are for Yate's chi‐square test. [file BJD-173-1471-s001.docx]

**Supplementary table 1**

Cutaneous phenotype of the CMN growth cohort (bold) and the endocrine study cohort (normal type), showing that the two cohorts are phenotypically similar.

| **Projected Adult**  **Size of largest CMN(cm)** | **N (%)** | **Site of largest CMN** | **N (%)** |
| --- | --- | --- | --- |
| <10 | **41(20);**7(15) | Face | **17(9);**2(4) |
| 10-20 | **35(17);**6(13) | Scalp | **20(10);**5(11) |
| 20-40 | **38(19);**9(19) | Trunk | **94(47);**24(51) |
| 40-60 | **30(15);**5(11) | Limb | **30(15);**5(11) |
| >60 | **51(25);**17(36) | Scalp/neck/trunk | **13(6);**2(4) |
| No single larger lesion | **4(2);**3(6) | Face/scalp | **15(7);**5(11) |
| Missing | **3(2);**0(0) | No single larger lesion | **4(2);**3(6) |
|  |  | Missing | **9(4);**1(2) |
| Total | **202(100);**47(100) | Total | **202(100);**47(100) |
|  |  |  |  |
| **Total number of other naevi at birth** | **N (%)** | **Total number of other naevi at enrolment** | **N (%)** |
| 0 | **30(15);**4(9) | 0 | **29(14);**4(9) |
| 1-10 | **50(25);**12(25) | 1-10 | **36(18);**7(15) |
| 11-20 | **31(15);**6(13) | 11-20 | **26(13);**7(15) |
| 21-50 | **29(14);**10(21) | 21-50 | **27(13);**3(6) |
| 51-100 | **16(8);**5(11) | 51-100 | **27(14);**8(17) |
| 101-200 | **10(5);**6(13) | 101-200 | **24(12);**10(21) |
| >200 | **4(2);**1(2) | >200 | **19(9);**6(13) |
| Missing | **32(16);**3(6) | Missing | **14(7);**2(4) |
| Total | **202(100);**47(100) | Total | **202(100);**47(100) |

**Supplementary table 2**

Phenotype and absolute values for ten patients with CMN who underwent OGTT. All concentrations other than glucose and insulin were only measured at baseline. x0, x30, x60, x90 represent values at time zero, 30, 60 and 90 minutes respectively from ingestion of standard glucose dose.

| **ID** | **Gender (female =1)** | **Decimal age (yrs)** | **BMI (centile)** | **BMI SDS** | **Glucose X0 (mmol/l)** | **Glucose X30 (mmol/l)** | **Glucose X60 (mmol/l)** | **Glucose X90 (mmol/l)** | **Insulin X0 (mU/l)(pmol/l)** | | **Insulin X30 (mU/l)** | **Insulin X60 (mU/l)** | **Insulin X90 (mU/l)** | **Inhibin (pg/ml)** | **LH (IU/l) (0.5-25.0)** | **FSH (IU/l) (3.5-10.0)** | **IGF-I (ng/ml) (94-501)** | **IGF-BP-3(mg/L)** |
| --- | --- | --- | --- | --- | --- | --- | --- | --- | --- | --- | --- | --- | --- | --- | --- | --- | --- | --- |
| 1 | 1 | 14.88 | 22.1 (50-75) | 0.8 | 4.6 | 8.2 | 6.7 | 3.8 | 11.5 | 52.2 | | 79.4 | 32.1 | 92.1 (14-362) | 1.3 | 4.8 | 374 | 7.33 (3.4-9.5) |
| 2 | 1 | 15.52 | 21.5 (50-75) | 0.464 | 4.8 | 7.3 | 5.9 | 5 | 29.6 | 81.6 | | 71.7 | 61 | 102.5 (14-362) | 3.2 | 6.2 | 214 | 4.78 (3.4-9.5) |
| 3 | 1 | 16.28 | 23.2 (75-90) | 0.882 | 4.1 | 5.8 | 4.8 | 5.3 | 5.5 | 23.2 | | 39.6 | 41 | 89.8 (14.362) | 3.8 | 6.2 | 120 | 4.73 (3.2-8.7) |
| 4 | 1 | 14 | 19.8 (50-75) | 0.158 | 4.6 | 7.7 | 5.3 | 5.8 | 10.7 | 158 | | 46.2 | 62.9 | 44.5 (14-362) | 2.9 | 4.5 | 296 | 4.9 (3.3-10.0) |
| 5 | 0 | 16.49 | 29.7 (97-99.5) | 2.269 | 4.4 | 8 | 8.5 | 6.6 | 24.6 | 183 | | 252 | 94.6 | 156.6 (74-470) | 4.5 | 2.9 | No result | No result |
| 6 | 1 | 12.05 | 25.7 (97-99.5) | 2.169 | 4.5 | 7.1 | 5.9 | 5.2 | 29.3 | 120 | | 138 | 86.2 | 47.7 (14-362) | 0.6 | 3.8 | 152 | 6.24 (3.1-9.5) |
| 7 | 1 | 15.94 | 25.5 (90-97) | 1.527 | 5.7 | 6.9 | 10.5 | 8.6 | 32.1 | 74 | | 144 | 134 | 48.7 (14-362) | 6.9 | 5.5 | 238 | 5.1 (3.4-9.5) |
| 8 | 0 | 16.33 | 22.2 (50-75) | 1.182 | 5.2 | 9.3 | 8.2 | 5.9 | 23.2 | 63.5 | | 198 | 124 | 209.3 (74-470) | 4.4 | 2.3 | 194 | 5.85 (3.4-9.5) |
| 9 | 1 | 12.35 | 19.3 (50-75) | 0.223 | 4.6 | 6.3 | 6.1 | 6 | 15 | 46.3 | | 50.4 | 50 | 104.9 (14-362) | 1.9 | 4.4 | No result | No result |
| 10 | 0 | 8.16 |  |  | 4.3 | 8.8 | 6.2 | 7.0 | 42.9 | >300 | | >300 | >300 | No result | No result | No result | No result | No result |

**Supplementary table 3**

Raw data from whole body composition analysis using DXA scanning of 25 patients with CMN. SDS = standard deviation score; Wt = weight; Ht = height; DX = dual Xray; BMC = bone mineral content; FM = fat-mass; FFM = fat-free mass. One patient was too young for DXA standard deviation score reference cohort data.

| **ID** | **Sex M=1** | **Age** | **Ht SDS** | **Wt SDS** | **BMI SDS** | **DX fat mass** | **DX Lean** | **DX BMC** | **DX FFM** | **DX FM SDS** | **DX FFM SDS** |
| --- | --- | --- | --- | --- | --- | --- | --- | --- | --- | --- | --- |
| CMN1 | 0 | 14.95 | -0.40 | 0.22 | 0.61 | 15.62 | 36.08 | 2.44 | 38.53 | 0.11 | -0.16 |
| CMN2 | 0 | 15.77 | -0.24 | 0.66 | 0.98 | 21.26 | 37.27 | 2.19 | 39.46 | 0.68 | -0.17 |
| CMN3 | 1 | 16.03 | 0.61 | 2.94 | 2.88 | 46.73 | 53.01 | 2.48 | 55.49 | 2.31 | 0.46 |
| CMN4 | 1 | 12.29 | -0.36 | -0.96 | -1.29 | 5.20 | 26.66 | 1.25 | 27.90 | -0.68 | -1.36 |
| CMN5 | 1 | 6.95 | -1.03 | -1.03 | -0.53 | 2.55 | 16.49 | 0.75 | 17.24 | -0.58 | -1.25 |
| CMN6 | 0 | 14.44 | 1.43 | 1.36 | 0.90 | 22.75 | 38.31 | 2.75 | 41.06 | 1.03 | 0.53 |
| CMN7 | 0 | 11.61 | 0.33 | 1.88 | 2.20 | 24.82 | 29.94 | 1.72 | 31.67 | 1.66 | 0.39 |
| CMN8 | 0 | 15.51 | -1.05 | 0.48 | 1.21 | 24.07 | 32.03 | 1.79 | 33.82 | 1.00 | -1.32 |
| CMN9 | 1 | 12.37 | 0.11 | 1.31 | 1.69 | 20.54 | 28.12 | 1.58 | 29.70 | 1.54 | -1.00 |
| CMN10 | 1 | 11.97 | 1.79 | 0.81 | -0.17 | 11.07 | 30.77 | 1.83 | 32.60 | 0.67 | 0.01 |
| CMN11 | 0 | 10.83 | -0.77 | -0.21 | 0.22 | 8.41 | 24.07 | 1.09 | 25.15 | -0.39 | -0.76 |
| CMN12 | 1 | 15.91 | 1.45 | 1.37 | 0.94 | 21.13 | 50.70 | 3.25 | 53.95 | 1.22 | 0.32 |
| CMN13 | 0 | 9.10 | 1.35 | 1.07 | 0.65 | 11.67 | 22.19 | 1.13 | 23.32 | 0.70 | -0.06 |
| CMN14 | 1 | 7.09 | 0.84 | 0.88 | 0.55 | 4.64 | 20.03 | 0.92 | 20.95 | 0.63 | 0.28 |
| CMN15 | 1 | 11.73 | -1.75 | -0.96 | 0.11 | 4.72 | 25.11 | 1.44 | 26.55 | -0.77 | -1.25 |
| CMN16 | 0 | 5.65 | 0.48 | -0.23 | -0.82 | 3.24 | 14.42 | 0.76 | 15.18 | -0.49 | -0.45 |
| CMN17 | 0 | 6.32 | 1.18 | 1.11 | 0.64 | 8.33 | 15.31 | 0.85 | 16.16 | 0.95 | -0.57 |
| CMN18 | 0 | 8.58 | -1.18 | 0.02 | 0.83 | 6.63 | 19.73 | 0.80 | 20.53 | -0.26 | -0.68 |
| CMN19 | 1 | 7.93 | -0.09 | 1.76 | 2.37 | 11.76 | 20.58 | 1.19 | 21.77 | 2.00 | 0.03 |
| CMN20 | 0 | 5.70 | 0.10 | 0.99 | 1.22 | 6.02 | 15.52 | 0.73 | 16.25 | 0.64 | 0.06 |
| CMN21 | 0 | 3.23 | 0.59 | 0.20 | -0.25 | 2.26 | 11.79 | 0.44 | 12.23 | Too young for ref data | |
| CMN22 | 0 | 5.48 | 0.48 | 0.28 | -0.07 | 2.76 | 15.79 | 0.72 | 16.52 | -0.70 | 0.40 |
| CMN23 | 1 | 5.38 | -1.46 | -0.50 | 0.74 | 2.17 | 15.39 | 0.63 | 16.02 | -0.77 | -0.65 |
| CMN24 | 1 | 7.76 | 1.52 | 1.75 | 1.42 | 8.43 | 23.12 | 1.07 | 24.19 | 1.51 | 1.03 |
| CMN25 | 1 | 6.25 | -0.31 | -0.55 | -0.56 | 1.70 | 16.92 | 0.72 | 17.63 | -1.44 | -0.57 |

**Supplementary figure 1**

Upper panel: Histograms showing absolute birth weight (kg) in females and males and occipito-frontal head circumference standard deviation score (SDS) for full-term infants in the cohort with CMN


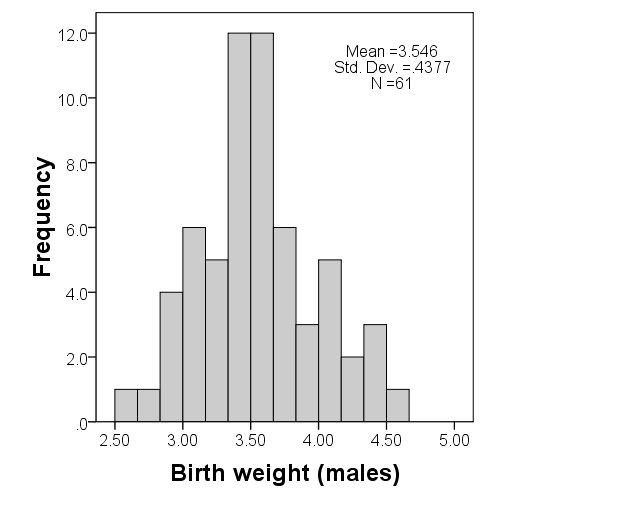

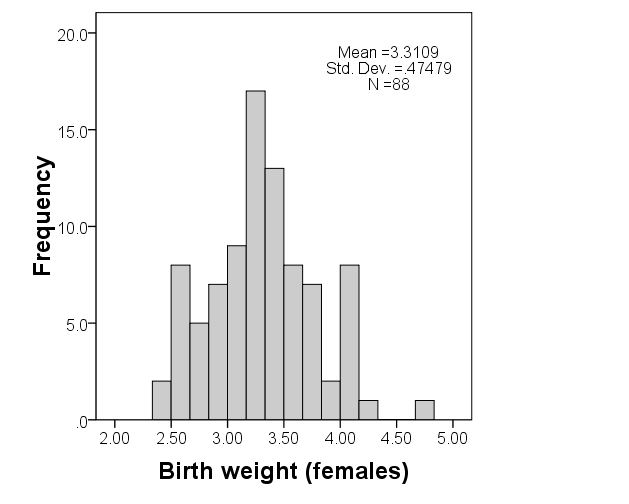
Lower panel: Comparison of method of onset of labour and delivery between the CMN cohort and national statistics 2009-1010. Numbers for induction of labour do not include elective caesarean section. P values are for Yate’s chi-square test.


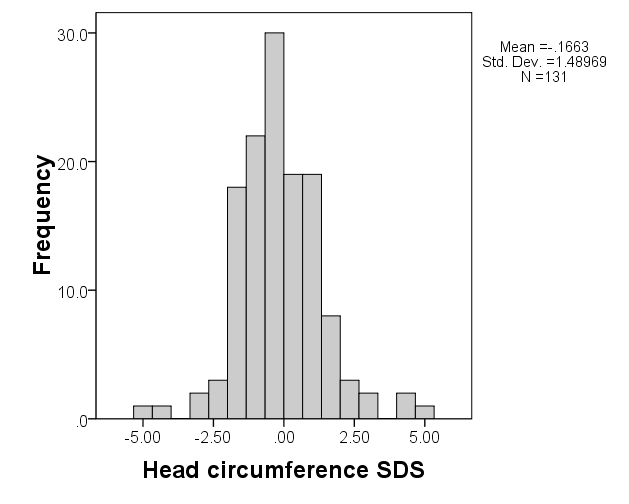


|  | **Labour Induced** | **Vaginal delivery** | **Instrumental delivery** | **Caesarian section** | **Unknown method** |
| --- | --- | --- | --- | --- | --- |
| **CMN cohort n (%)** | 35/134 (26.1) | 90/157  (57.3) | 18/157  (11.5) | 47/157  (29.9) | 2/157  (1.3) |
| **UK HES n (%)** | 53,897/  652,377 (8.2) | 396,702/  652,377  (60.8) | 78,831/  652,377  (12.1) | 157,589/  652,377  (24.2) | 16,636/  652,377  (2.6) |
| **p value** | **<0.0001** | 0.4166 | 1.0000 | 0.10991 |  |
